# Cantú Syndrome: A Poorly Understood Multi-Organ Disorder

**DOI:** 10.3390/ijms27146323

**Published:** 2026-07-16

**Authors:** Shuijing He, Dan Hu, Colin G. Nichols, Yan Huang

**Affiliations:** 1 Department of Cardiology and Cardiovascular Research Institute, Renmin Hospital of Wuhan University, Wuhan 430060, China; heshuijing2001@163.com (S.H.); rm002646@whu.edu.cn (D.H.); 2 Hubei Key Laboratory of Cardiology, Wuhan 430060, China; 3 Center for the Investigation of Membrane Excitability Diseases (CIMED), Washington University School of Medicine, St. Louis, MO 63110, USA; cnichols@wustl.edu; 4 Department of Cell Biology and Physiology, Washington University School of Medicine, St. Louis, MO 63110, USA

**Keywords:** Cantú syndrome, *ABCC9*, *KCNJ8*, ATP-sensitive potassium channel, congenital hypertrichosis, channelopathy

## Abstract

Cantú syndrome (CS) comprises a group of rare multisystem and multi-organ diseases characterized by congenital hypertrichosis, facial dysmorphism, cardiomegaly, and skeletal abnormalities, as well as other clinical manifestations. The current understanding of CS is limited. It is easy to misdiagnose it due to its diverse clinical manifestations and a lack of awareness of the condition. Currently, it is thought that this disorder is inherited in an autosomal dominant manner and caused by mutations in the ATP-sensitive potassium (K_ATP_) channel, which plays a vital role in both cardiovascular diseases and diabetes. There are currently no effective or specific treatments for CS, and there is a lack of research on the mechanisms of and therapy for this disease. Therefore, it is essential to raise awareness and educate people on CS. Herein, we summarize reported knowledge about CS, including its epidemiology, definition, clinical features, diagnosis, and treatment.

## 1. History and Epidemiology

Cantú syndrome (CS) (OMIM 239850) was first reported in Mexico in 1982 by Cantú et al. [1]. The initial report described four cases of macrosomia born with congenital hypertrichosis and multiple system involvement such as heart and skeletal abnormalities. Two of them are siblings, and the three families are not related to each other, which led to the initial belief that the disease was inherited in a recessive pattern.

Since then, sporadic cases have been discovered [2,3,4,5,6,7]. By early April 2026, more than 150 cases have been reported worldwide [5,8,9,10,11,12,13,14,15,16,17,18]. Most of the reported cases were Caucasian, and before 2012, the majority were Mexican [11]. We do not think that this distribution is regular or reflects the law of CS prevalence. Since 1982, although a few researchers have studied this disease, the early recognition, diagnosis, and case registration of CS have remained suboptimal in clinical practice.

In 1999, Robertson et al. conducted a segregation analysis based on all reported families and concluded that CS was unlikely to be inherited in an autosomal recessive manner [19]. Lazalde et al. first reported a case of male transmission in 2000 [20] and suggested that autosomal dominant inheritance was more likely than recessive inheritance, with CS potentially explained by parental gonadal mosaicism. Then it was determined that CS was inherited in an autosomal dominant pattern.

The early recognition and diagnosis of CS are mostly confined to clinical symptoms. However, the most prominent manifestations, including facial dysmorphism, congenital hypertrichosis, and cardiovascular and skeletal abnormalities, are not unique to CS. This has resulted in the misdiagnosis of CS with other diseases, like Zimmermann–Laband syndrome, lysosomal storage diseases, and acromegaly. Studies related to CS have increased progressively, and researchers have begun to conduct in-depth molecular genetic exploration. Studies have suggested that dominant missense mutations in *ABCC9* cause CS [21,22]. A mutation in the *KCNJ8* gene has also been found to cause the same symptoms [9,23,24]. This breakthrough sheds light on the nature of CS and distinguishes it from other diseases with which it has clinical overlap. At the same time, there have also been many retrospective cases without *ABCC9* and *KCNJ8* mutation-related tests, but they also showed similar clinical symptoms, which could be considered missed CS [12]. Some patients suspected of CS who did not have a pathogenic variant identified in *ABCC9* may carry a *KCNJ8* variant or require alternative diagnoses or may still have an undetected variant that causes CS [21,22,24]. In CS patients who have undergone exome sequencing, all gene mutations observed were located in the *ABCC9* or *KCNJ8* gene, which encodes the ATP-sensitive potassium (K_ATP_) channel [25,26].

CS is indeed rare. Most cases have been sporadic or familial, except for those in the International Cantú Syndrome Registry [2] established and led by an expert team from Washington University School of Medicine in St. Louis, Missouri, and Utrecht University in the Netherlands in 2012, which summarized phenotypic characteristics and associated genotypes in 74 individuals [11]. Also, there is a lack of international epidemiological investigations of this disease, and the unclear understanding of the disease and misdiagnosis seriously affect CS incidence statistics and research progress. Although no international consensus has been made on the genetic analysis and diagnosis procedure of CS, it is now possible to define, diagnose, and even treat CS on a molecular basis with advances in gene detection and gene treatment technology, greatly reducing the rates of misdiagnoses and missed diagnoses that were based only on clinical symptoms and the exclusion method.

## 2. Molecular Genetics and Pathophysiology

**Figure 1:**
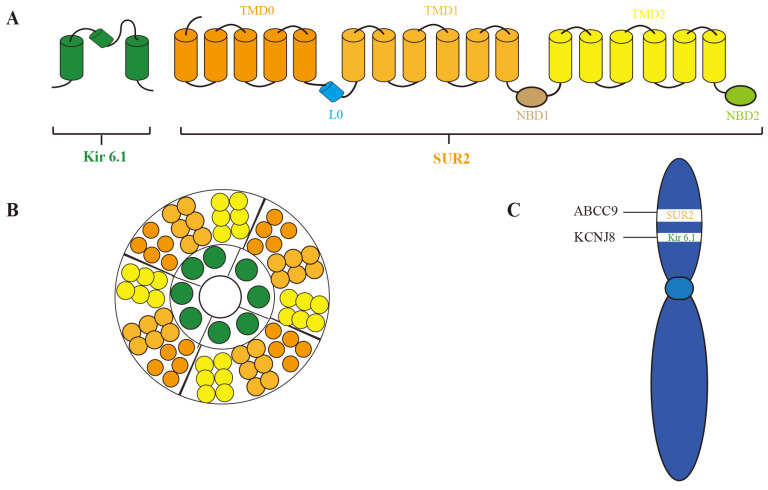
Kir6.1/SUR2 channel genes and structure. (**A**) The structure of Kir6.1 and SUR2. (**B**) The ATP-sensitive potassium (K_ATP_) channel is a hetero-octameric complex of four Kir6.x and four SURx subunits. (**C**) Chromosome 12-NC_000012.12. *KCNJ8* and *ABCC9* are located on human chromosome 12p12.1.

The K_ATP_ channel is a macromolecular complex composed of four inwardly rectifying K^+^ channel (Kir6.1 or Kir6.2) subunits forming a central pore surrounded by four peripheral regulatory sulfonylurea receptor (SUR) subunits (SUR1, SUR2A or SUR2B). The structure of the K_ATP_ channel can differ greatly from tissues and perform different functions because Kir6.x and SURX are freely assembled to form functional K_ATP_ channels [27]. The genes *ABCC9* and *KCNJ8* encode the SUR2 and Kir6.1 subunits, respectively. Gain-of-function (GoF) mutations in *ABCC9* and *KCNJ8* lead to CS [9,28]. Kir6.1 consists of proteins with two membrane-spanning α-helixes. The SUR2 subunit has 17 transmembrane regions, arranged in three domains, TMD0, TMD1, and TMD2, and two nucleotide-binding domains (Figure 1). To date, it has not been fully determined how the overactivity of the channels in which these two subunits are located leads to the variable clinical symptoms of CS and the related pathophysiology.

CRISPR/Cas9 genome engineering was used to introduce human disease-associated mutations into *ABCC9* and *KCNJ8* in mice and zebrafish to establish CS models [28,29,30]. SUR2[A478V], SUR2[R1154Q], and Kir6.1[V65M] mutant mice and SUR2[C1043Y], SUR2[G989E], and Kir6.1[V65M] zebrafish exhibited cardiac hypertrophy like CS patients, reproducing the key cardiovascular features of the disease. Furthermore, secondary CS cardiovascular features including vascular dilation, hypotension, and cerebrovascular diastole were reproduced. These results all suggest a causal relationship between the introduced mutations and the clinical symptoms of CS. For patients with different gene phenotypes, the severity of disease can be different. Even the same genetic phenotype can present different clinical symptoms [30,31]. Changes in different domains of the same subunit can result in differences in symptoms, and even small biophysical effects can lead to changes. Therefore, it is difficult to evaluate the occurrence and development of CS through the genotype alone. Further studies are needed to determine the direct relationship between phenotypes and genes. To date, 44 CS-related gene mutations have been identified in *ABCC9* and *KCNJ8*, corresponding to 47 reported protein alterations (Table 1). During data collection, we observed that more than 90% of the reported pathogenic variants are located in *ABCC9*, with SUR2[R1154Q] and SUR2[R1154W] being the most frequently reported.

Research and speculation on how mutations result in each clinical symptom are increasing [28,31,32,33]. It was demonstrated that symptom severity is not directly correlated with the sensitivity of K_ATP_ channels. Although the SUR2A (S1054Y) variant was predicted to be highly pathogenic, it partially attenuated the GoF by decreasing the membrane expression level of the channel protein [34]. Although Kir6.1 mutant mouse cardiomyocytes showed an essentially normal K_ATP_ channel character, the mouse heart maintained hypertrophy, consistent with the symptoms observed in patients [29]. Moreover, cardiac hypertrophy and increased contractility were more pronounced in Kir6.1 mutant mice than in SUR2 mutant mice, which may be related to the continuous activation of the Renin-Angiotensin-Aldosterone System (RAAS), increased blood volume or valve defects caused by chronic vasodilation and lower blood pressure [35]. Meanwhile, although the K_ATP_ GoF of vascular smooth muscle should theoretically lead to a decrease in peripheral resistance and cardiac output, increased cardiac output has been observed in both CS patients and experimental mice. This is hypothesized to be associated with the activation of the RAAS, adrenergic or parallel signaling pathways, and an enhanced L-type Ca^2+^ current in cardiomyocytes, as well as contractility. Some other authors suggest that mitochondrial Ca^2+^ overload and ROS generation, which subsequently leads to nitric oxide consumption and peroxynitrite formation, cause endothelial dysfunction in mice with CS [35]. This also indicates that it is inappropriate to routinely treat CS patients with hypertrophic cardiomyopathy or dilated cardiomyopathy and provides a new idea for the treatment of CS [36].

K_ATP_ channels containing Kir6.1 and SUR2 have also been found in the dermal papilla and sheath of human hair follicles and may control cell proliferation and hair growth. Hypertrichosis in patients with CS is considered to be caused by gene mutations that stimulate the opening of K_ATP_ and stimulate dormant hair follicles into the growth phase [37,38]. But the exact mechanism of action is not clear.

Pulmonary hypertension in CS patients does not appear to be consistent with the long-held view that it results from reduced potassium channel activity. This may be related to the activation of the RAAS or congenital cardiovascular structural abnormalities in CS, such as patent ductus arteriosus (PDA), aortic root dilatation, and bronchopulmonary dysplasia due to extreme preterm birth [35].

K_ATP_ channels are also expressed in the visceral smooth muscle, including the gastrointestinal tract. The Kir6.1 homozygous offspring of Kir6.1 heterozygous hybrid mice demonstrated a significant increase in mortality and sustained weight loss before death; intestinal distention was found at autopsy, suggesting impaired gastrointestinal function [29]. The experimental results showed that the inner intestinal neuron activity of the mice was not damaged, and K_ATP_ directly regulated contractile force through intestinal smooth muscle. At the same time, the decline in basic intestinal contractile force and muscle tone varied with changes in the mutant molecule [39]. This is also consistent with the symptoms of intestinal motility abnormalities observed in patients with CS.

Migraine, epilepsy and other symptoms in some patients with CS are thought to be related to cerebral vascular dilation, increased blood flow and a series of physiological changes secondary to K_ATP_ channel changes [40]. Lymphedema is considered to be due to the GoF of K_ATP_, which leads to a significant decrease in the contractile force of lymphatic smooth muscle and the failure of lymphatic fluid pumping [41]. Skeletal abnormalities may occur in parallel with cardiovascular manifestations; although Kir6.1 is only a minor component of K_ATP_ in skeletal muscle, its mutation causes serious fiber atrophy, damage to skeletal muscle integrity, and inflammatory cell infiltration. This suggests that the role of Kir6.1 needs to be further studied and that non-skeletal muscle cell mechanisms may play a key role [42]. Abnormalities of connective tissue and the immune system have been reported in CS-related cases [11], but whether they are directly related to changes in ion channels and their specific mechanisms remain unclear.

It is reasonable, though not fully supported, to predict clinical symptoms according to the GoF of the K_ATP_ channel alone. A series of subsequent reactions and compensatory mechanisms leads to complex clinical symptoms in patients with CS. When considering the pathophysiological process of an organ, it is necessary to consider not only the expression and effect of mutations in the organ but also the contribution of other mechanisms to its pathological changes.

**Table 1:** *ABCC9* and *KCNJ8* variants reported in Cantú syndrome (CS) patients.

Gene	Nucleotide Change	Protein Change	References
*ABCC9*	c.178C>T	p.His60Tyr	[21]
	c.621C>A	p.Asp207Glu	[21]
	c.1138G>T	p.Gly380Cys	[21]
	c.1295C>T	p.Pro432Leu	[21]
	c.1433C>T	p.Ala478Val	[21,22,32,33]
	c.3058T>C	p.Ser1020Pro	[21]
	c.3116T>C	p.Phe1039Ser	[21]
	c.3128G>A	p.Cys1043Tyr	[22]
		p.Cys1050Phe	[43]
	c.3161C>A	p.Ser1054Tyr	[21,43]
	c.3347G>A	p.Arg1116His	[21]
	c.3346C>T	p.Arg1116Cys	[21]
	c.3460C>T	p.Arg1154Trp	[44]
	c.3461G>A	p.Arg1154Gln	[21,22,32,33]
	c.3605C>T	p.Thr1202Met	[21,22,33,45]
	c.4039C>T	p.Arg1347Cys	[46]
	c.4385C>G	p.Ala1462Gly	[47]
	c.3014A>T	p.His1005Leu	[11]
	c.4469T>A	p.Val1490Glu	[11]
	c.3796G>A	p.Val1266Met	[11]
	c.3052T>G	p.Trp1018Gly	[11]
	c.3345G>A	p.Arg1116His	[11]
	c.881G>A	p.Gly294Glu	[11]
	c.4480G>A	p.Ala1494Thr	[11]
	c.3704C>T	p.Ser1235Phe	[11]
	c.3056C>A	p.Thr1019Lys	[11]
	c.3618C>A	p.Asn1206Lys	[11]
	c.3460C>G	p.Arg1154Gly	[11]
	c.2444G>C	p.Gly815Ala	[11]
	c.2378A>T	p.Asp793Val	[11]
	c.3055_3056delinsGA	p.Thr1019Glu	[11]
	c.3618C>G	p.Asn1206Lys	[11]
	c.3345C>G	p.Arg1116Gly	[11]
	c.4040G>T	p.Arg1347Leu	[11]
	c.2954A>C	p.Tyr985Ser	[28]
	c.2440G>T	p.Gly814Trp	[48]
	data not available	p.Met1060Ile	[28]
	c.2965G>A	p.Gly989Arg	[49]
	c.2966G>A	p.Gly989Glu	[49]
	c.3164_3173delinsC	p.Leu1055_Glu1058delinsPro	[49]
	c.2438G>C	p.Ser813Thr	[50]
	c.3058T>G	p.Ser1020Pro	[11]
		p.Gly989Glu	[28]
*KCNJ8*	c.193G>A	p.Val65Met	[23]
	c.526T>A	p.Cys176Ser	[24]
	c.565G>A	p.Glu189Lys	[10]
	c.994G>A	p.Glu332Lys	[9]

## 3. Clinical Characteristics

**Figure 2:**
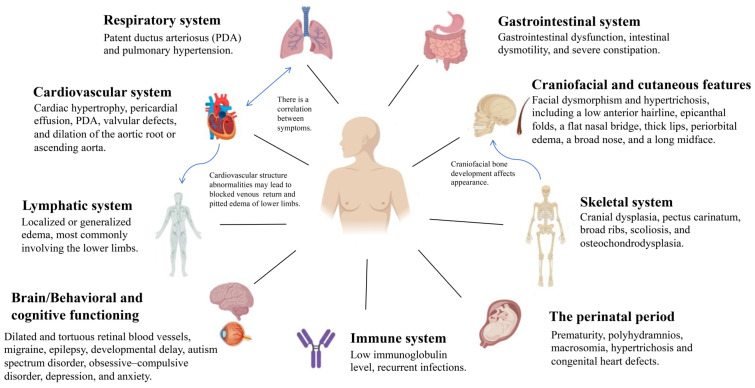
Multisystem pathologies in CS.

Since the GoF mutations of the *ABCC9* and *KCNJ8* genes lead to CS and are expressed in a wide range of tissues, CS involves multiple systems and organs and produces diverse symptoms (Figure 2). A few cases have been reported since 1982, involving various clinical symptoms that can change with age. There is currently no available evidence that supports a significant clinically relevant genotype–phenotype correlation for variants of *ABCC9* or *KCNJ8*.

Although most patients have hypertrichosis, facial dysmorphology and cardiovascular abnormalities, there can be large differences in these characteristics even within families.

### 3.1. Craniofacial and Cutaneous Features

Facial abnormalities and congenital hypertrichosis often provide important clues in the initial identification of the disease and are present in almost every CS patient. The former is usually evident at birth, while the latter is usually present within the first year of life. The facial features in these cases are distinctive and coarse in appearance, including a low frontal hairline, epicanthus folds, a flat bridge of the nose, thick lips, swollen eyelids, a wide nose, and facial lengthening. With age, the face lengthens further, along with the appearance of a flatter nose, sharper chin and fuller lips. The forehead and the roof of the mouth are more prominent than the middle of the face [8]. In partial cases, hypertrichosis is systemic, with thick hair covering the forehead and cheeks, arms, legs, and back. The face also shows characteristic thick eyebrows, eyelashes, and so on. Forehead hair tends to decrease with age.

### 3.2. Cardiovascular System

Cardiovascular involvement is highly prevalent and represents the major source of morbidity, including cardiac enlargement, pericardial effusion, PDA, valve defects, and aortic root or ascending aorta dilatation [51]. Despite the enlarged chamber of the heart in CS, cardiac function is normal, contractility is enhanced, and the heart is in a high output state. Heart failure is rare. However, whether the risk of heart failure increases with age warrants investigation [52]. At the same time, patients’ blood vessels in other parts of the body are abnormal, showing characteristics such as decreased resistance, dilation and bending. These vascular abnormalities lead to symptoms in the corresponding tissues and systems.

### 3.3. Skeletal System

CS is also known as “hairy osteochondroplasia” because it can cause skeletal abnormalities including cranial prolapse [45], coarse facial features, broad ribs, scoliosis, osteochondrodysplasia [19]. Skeletal abnormalities generally have a limited impact on daily life; therefore, not all patients undergo imaging, and reports of prevalence are limited.

### 3.4. Lymphatic System

Lymphedema occurs in half of CS patients [11,53]. Systemic edema at birth typically resolves spontaneously with age. In puberty or early adulthood, local edema often appears in the calf, particularly among females. The *ABCC9* variant p.(Leu1055_Glu1058delinsPro) was found in a patient diagnosed with idiopathic lymphedema and introduced into the equivalent site of rat SUR2A, with the K_ATP_ channel’s GoF. At the same time, a retrospective analysis found that this patient’s symptoms overlapped with those of CS patients. Lymphedema may be one of the earliest clinical symptoms in some patients with CS, but more studies are needed to confirm this hypothesis [54]. Meanwhile, abnormal cardiovascular structure may also lead to blocked venous return and pitted edema of the lower limbs, which needs to be identified.

### 3.5. Respiratory System

Pulmonary hypertension in CS patients has traditionally been reported predominantly in infants and young children and was largely attributed to underlying congenital heart defects, including mitral valve stenosis and regurgitation, as well as associated respiratory malformations and multisystem involvement. A recent study indicated that the constitutive activation of SUR2A/Kir6.1 channels leads to systemic vasorelaxation and hypotension, which in turn triggers compensatory cardiac hypertrophy and hypercontractility, ultimately contributing to the development of pulmonary hypertension [55]. Pulmonary vascular abnormalities and pulmonary hypertension may be related to mutual shortness and respiratory distress as well as exercise intolerance in some children with CS [56]. Most CS cases with pulmonary hypertension have mild symptoms and improve with age. However, those with serious pulmonary hypertension at birth have a poor prognosis. One female infant with heterozygous c.4385C>G (p.Ala1462Gly) had serious pulmonary hypertension and died of cor pulmonale and sepsis [47]. Another patient was reported to have pulmonary hypertension at birth and needed continuous positive airway pressure ventilation, which was reversed by glibenclamide [14].

### 3.6. Brain

Previous studies have focused on cerebrovascular malformations. The diffuse dilation of cerebral blood vessels and distorted cerebral blood vessels are thought to be related to migraine and epilepsy in some patients with CS. At the same time, changes in subcortical white matter were observed in brain magnetic resonance imaging (MRI) examination, which may be related to long-term cerebral vascular diastole and changes in cerebral vascular reserve. One study found that patients with CS exhibit increased cerebral blood flow without a corresponding significant elevation in cerebral oxygen utilization [57]. Dilated and curved blood vessels in the retina were also observed [58].

### 3.7. Gastrointestinal System

Some patients have gastrointestinal dysfunction, showing different symptoms and degrees. Some of them develop gastroesophageal reflux in infancy and require tube feeding [8]. A smaller number of patients have intestinal dysmotility and severe constipation [39].

### 3.8. Immune System

It has been reported that some patients with CS have recurrent respiratory tract infection or severe infection, which is difficult to heal. Examination revealed low immunoglobulin levels. The types of immunoglobulins at low levels are not consistent; both IgA and IgG have been reported [8]. However, most patients have normal immune levels [11]. At the same time, hypoproteinemia can lead to symmetric edema of the lower limbs, which should be distinguished from lymphedema. The immune system needs further study in this context.

### 3.9. The Perinatal Period

The most common prenatal manifestation is prematurity and polyhydramnios. Neonates may present with macrosomia, hypertrichosis and congenital heart defects [6]. A previous study had reported that all newborns have hypertrichosis [2]. Growth parameters, including height, head circumference and weight, are higher than those of the general population. Overgrowth features are often observed at birth and persist throughout life [11]. Some patients exhibit hypotonia and reduced motor activity in early life. Developmental delays have also been reported, but persistent intellectual disability is rare [59].

### 3.10. Behavioral and Cognitive Function

There are two main aspects to CS’s impact on behavioral and cognitive function. One is caused by pathophysiological changes and their effects due to the K_ATP_ channels’ GoF. The other is the mental block caused by congenital hypertrichosis, facial deformities and other physical features with the burden of treatment expense. The former includes cerebral vascular abnormalities and emotional changes caused by frontal lobe lesions [8]. The latter, which includes anxiety and depression, is more common in men in early adulthood [59]. Some patients diagnosed with behavioral problems such as autism, obsessive–compulsive disorder, depression, and anxiety have been able to maintain normal and independent intellectual activity into adulthood despite delayed early development and can live and work normally.

Even patients with the same genetic mutation do not have identical emotional cognition. Emotional cognition also changes with age in the same patient. This indicates that it is also deeply influenced by family and society. It cannot be ruled out that CS patients are over-protected by their families and receive different examinations and treatments from their peers, which may lead to changes in their emotional cognition. At present, it is not clear whether the GoF of the K_ATP_ channel can directly lead to emotional problems such as cognitive impairment and anxiety. Therefore, it is necessary to conduct longitudinal studies on CS patients.

### 3.11. Other Infrequent Features

CS patients also have aortic aneurysm [45], abnormal oocyst development [60], pituitary abnormalities [8,61], connective tissue abnormalities such as skin laxity or wrinkling, and other rare symptoms. Pituitary abnormalities should be distinguished from congenital hypothyroidism. Endocrine abnormalities in CS have also received attention [17]. Whether there is a direct correlation between them and CS remains under study. Studies have also noted that lower voice pitch is a new phenotypic feature of CS. However, this is not the primary feature that draws our attention to patients with CS [49].

## 4. Clinical Evaluation

CS has a variety of symptoms, the degrees of which can be quite different. After CS is strongly suspected, the severity of different symptoms in patients should be evaluated before diagnosis and treatment. The assessment is based mainly on clinical symptoms, physical examination and laboratory examination. It is important to also rule out other causes of similar symptoms.

For follow-up treatment, it is best to evaluate the condition of patients in a separate system. Patients with a history of any organ disease and clinical manifestations should be considered for the corresponding system evaluation. We suggest that for patients diagnosed with CS, as many organ systems as possible should be evaluated. Patients often ignore the existence of some problems because of mild symptoms, which is a considerable obstacle to CS research, early diagnosis, and treatment.

The possibility that the corresponding symptoms result from other causes should be ruled out before each of the following systematic assessments.

### 4.1. Physical Examination

After obtaining a detailed medical family history of patients suspected of CS, targeted physical examinations need to be performed. The characteristic features are facial dysmorphism, hair growth, and cardiac and skeletal abnormalities. For fetuses, maternal comprehensive obstetric examinations are recommended to check whether the gravida’s abdominal circumference matches the gestational age. In neonates, attention should be paid to their growth and developmental milestones, with other physical assessments similar to those for adults.

### 4.2. Investigations

Appropriate laboratory tests should be selected according to the patient’s clinical manifestations, including diverse routine biochemical parameters and radiological assessment.

Hypoproteinemia can be initially evaluated by measuring total serum protein and albumin levels. Immunoglobulin profiling may be performed to determine the type and extent of immunoglobulin deficiency. It is important to note that some multisystem manifestations are relatively uncommon but may still be associated with CS. Therefore, targeted endocrine evaluations are recommended when it is clinically indicated, including an assessment of pituitary function and evaluation for potential connective tissue or reproductive system involvement. Although each assessment is independent, clinical severity and subsequent management need to be considered comprehensively.

Electrocardiogram (ECG) and echocardiography are recommended for the initial evaluation. Right heart catheterization can confirm pulmonary hypertension and assess vascular reactivity, but it is more invasive. ECG and echocardiography are recommended for the initial evaluation. Pulmonary artery pressure can be initially determined by echocardiography, while other possible causes can be ruled out by pulmonary function examination, arterial blood gas analysis, and chest computed tomography pulmonary angiography. The severity of respiratory symptoms can be determined in patients. Further cardiac magnetic resonance should be used to evaluate the cardiac structure and for differential diagnosis if needed.

Computed tomography (CT) and X-ray are primarily used to evaluate structural abnormalities, such as skeletal development status. CT and MRI may be used to assess lymphedema.

MRI and magnetic resonance angiogram (MRA) can be performed to evaluate neurological symptoms [62], especially for patients with migraines, epilepsy, hemiplegia and other diseases.

Fetal ultrasound is used to determine whether the fetus is macrosomic and the presence of polyhydramnios and cardiovascular dysplasia. However, if neither of their parents and no one in their family has been diagnosed with CS, it is difficult to consider the diagnosis of CS even if there may be signs on ultrasound. Amniocentesis can help in diagnosis.

### 4.3. Other Specialized Assessments

Lymphangiography can distinguish primary lymphedema from secondary lymphedema. However, these examinations are indirect diagnoses. Lymphatic vessel biopsy is needed to confirm that lymphedema is caused by the GoF of the K_ATP_ channel. However, this procedure is traumatic and causes many side effects, so it is not performed clinically at present.

Both infants and adults should be evaluated for their developmental status—physical, intellectual, and neuropsychological.

Patients presenting with any clinical manifestations are encouraged to undergo comprehensive evaluations of the relevant system, and the results should be considered collectively for overall clinical assessment.

## 5. Diagnosis

As of yet, there are no formal and internationally agreed-upon diagnostic criteria for CS. The most important thing for the diagnosis of CS is strong clinical evidence and gene detection [7,18]. Most cases have been reported under three conditions. One is when patients with CS seek medical attention due to cardiovascular abnormalities [63]. In other cases, neonates are identified immediately by their coarse facial features, hypertrichosis, or significant cardiac abnormalities such as PDA, ventricular septal defects, and patent foramen ovale. In one case, further information collected revealed that a neonate was born with macrosomia and that its mother was at risk for polyhydramnios during pregnancy. Further, some family members had a child or parent diagnosed with CS, so CS was highly suspected given the patient’s physical features. Based on this, after ruling out diseases such as Pompe and Zimmermann–Laband syndrome, a sequencing analysis was performed, and CS was confirmed.

Considering that the clinical manifestations of CS differ to varying degrees, early genetic testing should be considered for patients with a combination of the following symptoms: 1. Infants presenting with macrosomia, congenital hypertrichosis, coarse facial features, and congenital cardiac abnormalities. 2. Other unexplained cardiovascular abnormalities, such as myocardial hypertrophy, increased myocardial contractility, pericardial effusion, PDA, and hypertrichosis acromegaloid facial features disorder. 3. Other unexplained skeletal abnormalities such as cranial premature closure and/or thickening, rib widening, scoliosis, dysplasia of the pubic and ischial bones, obturator stenosis, and hip ectropion. 4. Craniofacial malformations of other causes were excluded, such as protruding forehead, longer middle, low nose bridge, enlarged nose, and thick lips. For patients with more prominent and typical symptoms, clinicians can include CS into the diagnostic scope for gene-targeted diagnostic detection.

## 6. Differential Diagnosis

Although the clinical features of CS are characteristic, no individual manifestation is sufficiently specific. The differential diagnosis of CS is primarily guided by the overlap of clinical features, such as overgrowth syndromes, growth and developmental delays, hypertrichosis, and congenital cardiovascular abnormalities. Based on this, several disorders should be considered differential diagnoses, as summarized in Table 2.

Hypertrichosis and coarse appearance are clinical manifestations of overlapping phenotypes of CS, acromegalic facial appearance syndrome, and hypertrichosis acromegaloid facial features disorder. For a long time, these three diseases were often distinguished by the presence of cardiovascular abnormalities and bone malformations, as well as the most prominent clinical symptoms. But as research progressed, it became clear that no particular clinical manifestation was specific to any particular disease [33,64]. Some scholars have suggested that the three diseases should be called *ABCC9*-related diseases [32]. However, it has been found that the gene mutations in *KCNJ8* can also cause corresponding symptoms. Therefore, under the premise of excluding other possible causes, combined with the results of genetic testing, it is now more common to believe that those above are not independent diseases but different phenotypes of CS [28,65]. Alternatively, CS can be considered to be a more serious clinical classification of Kir6.1/SUR2 GoF-related diseases.

## 7. Management

### 7.1. Symptomatic Treatment

There is currently no specific therapy for CS; symptomatic treatment is still the main option. That is, after the initial assessment, the patient’s symptoms are addressed (Table 3).

### 7.2. Targeted Therapies and Medications to Avoid

Although how the pathophysiological process of Kir6.1/SUR2 channel hyperactivity leads to the many characteristic symptoms of CS is not clear, the molecular mechanism has been gradually clarified. With the development of K_ATP_ channel research [73], K_ATP_ channel inhibitors have been shown to treat K^+^ channel-related diseases. Sulfonylureas, for example, glibenclamide, have been used as antidiabetic agents, which have effects not only on pancreatic K_ATP_ channels but also on cardiovascular K_ATP_ channels. Several reports [2,74,75] indicate that glibenclamide can be considered a treatment for CS, and the side effect of hypoglycemia is rare and can be alleviated by itself. At the same time, some studies [14] show that the effect of glibenclamide on SUR1 is stronger than that of SUR2. SUR2 (or Kir6.1)-specific blockers can be developed based on this. Certainly, more animal studies, as well as clinical trials, should be carried out before these K_ATP_ channel blockers can be used to treat CS.

There are also drugs to avoid based on molecular pathology, including diazoxide and minoxidil, which activate K_ATP_. The incorrect use of beta receptor blockers can worsen symptoms in patients with CS.

### 7.3. Genetic Counseling

The genetic and clinical evaluation of high-risk relatives of patients who have been diagnosed with CS is necessary. This can determine as early as possible whether a relative should be evaluated for CS-related symptoms. Problems such as heart structure and spinal development can also be treated early to prevent further development. At the same time, the systematic examination of family members is conducive to follow-up genetic counseling.

Since the inheritance pattern is autosomal dominant, it is recommended that patients with CS undergo molecular genetic testing, confirm their parents’ clinical and genetic status, and undergo reliable counseling for recurrence risk. The effects vary depending on the molecular basis. Genetic counseling for affected or at-risk young people—including understanding potential risks to offspring and reproductive options—is appropriate and necessary. The best time to determine genetic risk and discuss risk based on genetic testing is before pregnancy [2].

After pregnancy, prenatal testing may be provided with genetic counseling. Although the age and severity of the onset of CS in the fetus cannot be confirmed through the genetic phenotype alone, we still recommend prenatal genetic testing. It is necessary to carry out an ultrasound to detect macrosomia and polyhydramnios, as well as fetal echocardiography to assess fetal cardiovascular abnormalities. These inspections can be prepared in advance to reduce the risk of production; however, the early detection of some problems may be beyond the capacity of the family. After appropriate genetic counseling and multidisciplinary consultation, the family is left to make the reproductive decision.

Management during pregnancy requires attention to the mother’s own condition in addition to appropriate prenatal testing, especially the symptoms that were serious before pregnancy.

For CS patients in infancy, attention should be paid to their physiological and psychological development. In addition to symptomatic treatment, the systematic management of developmental delay is needed.

### 7.4. Long-Term Follow-Up

At present, follow-up data on CS patients remain limited, and the development of and changes in the disease are not well understood. We recommend systematic registration and regular follow-up for patients diagnosed with CS. The early detection and solution of problems are also conducive to CS research.

## 8. Conclusions

CS is a rare K_ATP_ GoF multisystem disorder caused by mutations in *ABCC9* and *KCNJ8*, for which the clinical spectrum is broad and often non-specific. At present, there is no targeted pharmaceutical treatment, and symptomatic treatment is the main treatment (Table 3).

A key unresolved issue is the unclear genotype–phenotype correlation, suggesting without a doubt that many patients with CS may be undiagnosed or misdiagnosed. Underestimating the incidence of CS has both hindered further research and prevented it from appearing in the public eye. Research on sulfonylureas may provide insights into targeted therapy. In addition, progress in CS research can prove the possibility of other ion channel diseases.

Future work should focus on improving diagnostic recognition, clarifying genotype–phenotype relationships, and exploring the targeted modulation of K_ATP_ channel activity to enable mechanism-based therapies.

## Figures and Tables

**Table 2:** Differential diagnosis of CS.

Disorder	Pathogenesis	Clinical Comparison with CS (Representative)		Reference
		Overlap	Distinctive	
Beckwith–Wiedemann syndrome	Genetic or epigenetic defects within the 11p15.5 region, including *CDKN1C* and *IGF2*, which are strong regulators of fetal growth.	Overgrowth and macroglossia.	Abdominal wall defects, neonatal hypoglycemia, lateralized overgrowth and predisposition to embryonal tumors.	[64]
Berardinelli–Seip congenital lipodystrophy	Pathogenic variants in *AGPAT2* or *BSCL2*, which disrupt the normal development and function of adipocytes and prevent the normal storage of fat in lipid droplets.	Cardiac abnormalities, such as cardiomegaly and acromegaloid facies.	Hypertriglyceridemia, hyperinsulinemia, hyperglycemia, hypoleptinemia, and diabetes mellitus.	[65]
Zimmermann–Laband syndrome	Pathogenic variants in *KCNH1*, *KCNN3* or *ATP6V1B2*.	Coarse facial features, overgrowth, hypertrichosis, skeletal abnormalities, and possible patent ductus arteriosus (PDA).	Intellectual disability and toenail hypoplasia.	[66]
Pompe disease	Pathogenic variant in *GAA*, localized on chromosome 17 and encoding for acid alpha-1,4-glucosidase.	Developmental delay, cardiac and skeletal abnormalities, macroglossia, and exercise intolerance.	Hepatomegaly, splenomegaly and progressive intellectual disability or neurologic deterioration in some persons.	[67]
Mucopolysaccharide accumulation	Pathogenic variants in 13 different genes, including *IDUA*, *IDS*, *SGSH*, and *NAGLU*, cause the lysosomal accumulation of glycosaminoglycans.	Coarse facial features, hirsutism, skeletal abnormalities and exercise intolerance.	Cognitive impairment, short stature, cloudy cornea, decreased vision, and hearing impairment.	[68]
Congenital hypothyroidism	Attributable either to pathogenic variants in different genes, including *TSHR*, *FOXE1*, *NKX2-1*, *PAX8* and *NKX2-5*, or to thyroid dysgenesis, iodine deficiency, and maternal anti-thyroid drugs, among others.	Coarse facial features, hypertrichosis, macroglossia and edema.	Severe and irreversible intellectual and physical developmental disabilities.	[61]
Acromegaly	Excess of human growth hormone, usually from a growth hormone-secreting pituitary adenoma.	Macrocephaly, coarse facial features, acral enlargement and soft tissue overgrowth.	Elevated GH/IGF-1 levels and pituitary adenoma.	[69]
Non-syndromic inherited cardiomyopathies	Pathogenic variants in genes that lead to cardiomegaly.	Manifestations are primarily limited to the cardiovascular system.	Absence of noncardiac findings.	[70]
Drug-induced Cantú-like phenotype	Pharmacological activation of K_ATP_ channels, such as minoxidil and diazoxide.	Most of the clinical manifestations overlap with those observed in CS, including coarse facial features and pericardial effusion.	Drug exposure history. Symptom improvement after drug withdrawal.	[71,72]

**Table 3:** Multisystem summary of CS.

System/Concern	Manifestations Frequently Observed	Evaluation	Treatment
Craniofacial and cutaneous features	Coarse facial features, hypertrichosis.	3D facial examination [62] and dermatologic assessment, endocrine and subsequent laboratory tests.	Do not require treatment unless patients have cosmetic concerns or psychosocial distress. Hair removal with a razor, depilatory creams or laser treatment. Attention to psychological needs.
Cardiac abnormalities	Cardiac hypertrophy, pericardial effusion, PDA, valve defects, aortic root or ascending aorta dilatation, among others.	Echocardiography and electrocardiogram (ECG), other tests if needed. Dynamic observation of development, such as aortic tumor enlargement, pericardial effusion and so on.	Review regularly, if patients have no obvious symptoms. Patients who need to be treated are dealt with by specialists according to their circumstances. For example, management of PDA follows standard cardiology practice and may require surgical or catheter-based closure during infancy or early childhood. CS patients should not be treated directly as in hypertrophic cardiomyopathy or dilated cardiomyopathy.
Skeletal abnormalities	Skeletal abnormalities, showing the typical “coarse” appearance of CS.	Comprehensive physical examinations and X-ray or computed tomography (CT) could be considered.	It is generally not treated. In cases of severe scoliosis, surgery may be considered.
Lymphedema	Local or systemic edema.	Ruling out other causes by CT or magnetic resonance imaging (MRI).	Pressure socks and limiting sodium intake.
Respiratory system	Structural abnormalities such as pulmonary hypertension and related clinical symptoms such as exercise intolerance.	ECG, echocardiography and CT are recommended.	Actively treat congenital cardiovascular abnormalities. Drugs for reducing pulmonary hypertension; for example, sildenafil can also be selected, or interventional and surgical treatment can be considered, so as to relieve clinical symptoms and increase activity tolerance.
Brain	Migraine and epilepsy associated with vascular abnormalities.	CT, MRI or magnetic resonance angiogram (MRA) could be considered according to their symptoms.	Corresponding specialized standards. If combined with migraine, neurologist can prescribe drugs for treatment.
Gastrointestinal system	Gastroesophageal reflux, constipation, intestinal dysmotility.	CT and gastroscopy and colonoscopy if necessary.	Tube feeding to obtain nutrition, appropriate drugs to adjust intestinal function, and if necessary, could be treated with surgery. Management of gastrointestinal dysmotility may include dietary modification and prokinetic agents under specialist supervision, like domperidone.
Immune system	Low immunoglobulin levels.	Laboratory testing.	Treatment is generally not required; intravenous immunoglobulin may be considered in selected patients.
Perinatal period	Polyhydramnios, macrosomia, congenital heart disease.	Maternal comprehensive obstetric examinations and fetal ultrasound. Neonatal physical examination is needed.	Communicate with family members, proactive symptomatic treatment before delivery and actively treat congenital heart disease of neonates.
Behavioral and cognitive functioning	Abnormal manifestations caused by pathophysiological changes and psychological and emotional abnormalities.	Assessment should be performed by a specialist.	Symptomatic treatment, and pay attention to the psychological problems of patients and their families. Early behavioral intervention.
Others	Pituitary gland, connective tissue, reproductive system and others.	Select investigations as needed.	Treated by their specialty according to the actual situation.

## Data Availability

No new data were created or analyzed in this study. Data sharing is not applicable to this article.

## Abbreviations

The following abbreviations are used in this manuscript:

CS	Cantú syndrome
K_ATP_	ATP-sensitive potassium
Kir	inwardly rectifying K^+^ channel
SUR	sulfonylurea receptor
RAAS	Renin-Angiotensin-Aldosterone System
PDA	patent ductus arteriosus
GoF	gain of function
ECG	electrocardiogram
MRI	magnetic resonance imaging
CT	computed tomography
MRA	magnetic resonance angiogram
